# Electronic Structure and d–d Spectrum of Metal–Organic Frameworks with Transition-Metal Ions

**DOI:** 10.1021/acs.jpcc.3c05025

**Published:** 2023-10-27

**Authors:** Ilya Popov, Dmitrii Raenko, Andrei Tchougréeff, Elena Besley

**Affiliations:** † School of Chemistry, University of Nottingham, University Park, Nottingham NG7 2RD, U.K.; ‡ A.N. Frumkin Institute of Physical Chemistry and Electrochemistry RAS, Moscow 119071, Russia

## Abstract

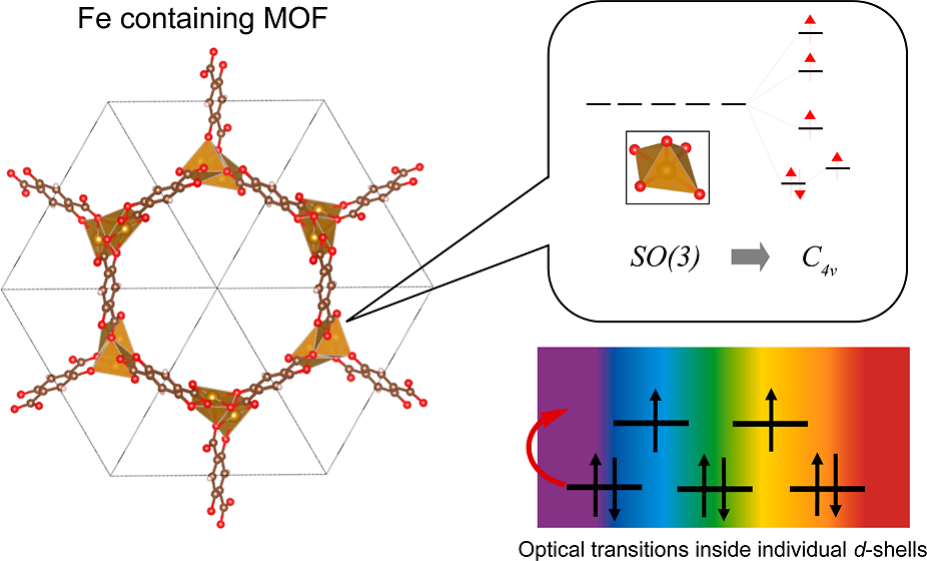

The electronic structure of metal–organic frameworks (MOFs) containing transition metal (TM) ions represents a significant and largely unresolved computational challenge due to limited solutions to the quantitative description of low-energy excitations in open d-shells. These excitations underpin the magnetic and sensing properties of TM MOFs, including the observed remarkable spin-crossover phenomenon. We introduce the effective Hamiltonian of crystal field approach to study the d–d spectrum of MOFs containing TM ions; this is a hybrid QM/QM method based on the separation of crystal structure into d- and s,p-subsystems treated at different levels of theory. We test the method on model frameworks, carbodiimides, and hydrocyanamides and a series of *M*-MOF-74 (M = Fe, Co, Ni) and compare the computational predictions to experimental data on magnetic properties and Mössbauer spectra.

## Introduction

Metal–organic frameworks (MOFs) are actively studied crystalline porous materials^1,2^ with a wide range of remarkable properties^3^ and applications which include gas storage,^4,5,6,7^ separation,^4,8−15^ and sensing,^16^ heterogeneous catalysis,^17,18^ biomedical imaging,^19^ and other areas. MOFs containing transition-metal (TM) ions represent a particularly interesting class since their complex electronic structure caused by the presence of open d-shells yields unique responses to the adsorption of small molecules,^20−28^ interaction with magnetic field,^29^ and temperature changes.^30,31^ Changes in optical, electrical, and magnetic properties of MOFs caused by the adsorption of guest molecules have been actively used in sensing applications.^20,25,32,33^ They can be associated with the spin-crossover phenomenon,^34^ in which spin of the ground state of a d-shell switches due to an external perturbation, in this case, due to rearrangement of the coordination sphere of TM upon adsorption. This is possible due to the presence in the MOF electronic structure of a low-lying excited state with a spin different from the spin of the ground state. Therefore, quantitative studies of the sensing behavior of MOFs containing TMs require an accurate theoretical description of the electronic structure featuring open d-shells, the spin and symmetry of the ground state, the multiplicity and energy of the low-energy multireference states of the d-shells, and the low-lying excitations inside an individual d-shell. In addition, the low-energy part of the d–d spectrum plays a crucial role in interpreting Mössbauer spectra of iron-containing MOFs and understanding the catalytic activity of TM-containing materials.^35−37^

Computational approaches to predicting the complex electronic structure of MOFs containing TMs focus their attention on different aspects of the problem. Periodic density functional theory (DFT) and its various combinations with high-level quantum chemical methods,^38−41^ explicitly taking into account the multireference nature of the electronic states of the d-shells,^42^ are often used to describe the ground-state properties of MOFs. The effective Hamiltonian of the crystal field (EHCF) method allows us to simultaneously describe the electronic structure of the ground state and the low-energy spectrum of the d-shells.^43−45^ This method, originally developed for finite chemical systems, has been extended recently to periodic solids and used to reproduce optical spectra of well-studied materials such as TM oxides with the rock-salt structure and TM impurities in the MgO matrix.^46^ This was made possible due to the computational efficiency and accuracy of the EHCF method which separates the total electronic wave function of a chemical system into the wave functions of the d- and s,p-subsystems following a *sui generis* QM/QM hybrid approach.^47^

In this work, we extend the EHCF method to describe the electronic structures of MOFs. We first tested the EHCF method on relatively small and experimentally well-studied compounds possessing the key features of MOFs and then described the electronic and magnetic properties of selected MOF structures. Carbodiimides and hydrocyanamides of TMs with the general formulas *M*NCN and *M*(HNCN)_2_ were chosen as suitable model systems. They contain structural components characteristic of MOFs such as TM ions and rodlike anions NCN^2–^ and HNCN^–^ with both σ-bonds and a delocalized π-system which play the role of organic linkers forming the frameworks. In addition, these systems are semiconductors, and their magnetic properties are known from experiment.^48,49^ After testing the EHCF approach on carbodiimides and hydrocyanamides containing various TMs (Fe, Co, and Ni), we apply it to study a series of MOF-74 frameworks.^50−52^ We calculate the magnetic moment of metal atoms, d–d spectrum, and temperature dependence of the quadrupole splitting in Mössbauer spectra of iron-containing compounds and compare the computational predictions to available experimental data.

## Computational Methodology

### Effective Hamiltonian of the Crystal Field Method for Periodic Systems

In this section, we give a brief introduction to the periodic EHCF method, while a complete description can be found in our previous work.^46^ The main idea of EHCF^43,45^ is to divide a space of one electronic state into d- and l-subspaces spanned by local atomic d- and s,p- orbitals, respectively, and to express the total wave function of a system as
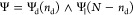
1where Ψ_d_ and Ψ_l_ are the many-electron wave functions built in the d- and l-subspaces, *n*_d_ is the number of electrons in the d-space, *N* is the total number of electrons in the system, and ∧ stands for the antisymmetric product. Once partitioning of the total wave function is performed, we employ the hybrid treatment of the d- and l-subsystems. The wave function of the highly correlated d-subsystem, Ψ_d_, is represented in the form prescribed by the configuration interaction (CI) method, while Ψ_l_ is approximated, within the Hartree–Fock–Roothaan approach, by a single Slater determinant on the basis of the Bloch sums of atomic valence sp-functions
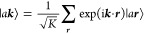
2here, |*a****r***⟩ are atomic (spin-)orbitals spanning the l-subspace and located in the ***r***-th unit cell of the crystal, ***k*** is a vector in the first Brillouin zone, and *K* is the number of unit cells in the model crystal defined within periodic boundary conditions. Describing the d-subsystem with the CI method avoids common convergence problems in the self-consistent field procedure, which are typically caused by the presence of open d-shells.

Electronic states involved in electron transfer between d- and l-subsystems and Coulomb interactions between them are taken into account through correction terms to the respective Hamiltonians, as described in refs (43) and (46), leading to the following effective Hamiltonian of the d-system
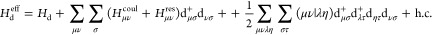
3where d_μσ_^+^ (d_μσ_) are the electron creation (annihilation) operators for the μ-th d-orbital, σ corresponds to the spin projection, (μν|λη) are the one-center two-electron Coulomb integrals inside the d-shell, *H*_d_ is a bare one-electron Hamiltonian of the d-system, *H*^coul^ and *H*^res^ describe Coulomb and resonance interactions of d-electrons with electrons and nuclei of the l-subsystem. *H*^coul^ is represented as a sum of the crystal field contribution^43,53^ and intra-atomic Coulomb repulsion of d-electrons and s,p-electrons of the same TM atom. The resonance term has the following form^46^
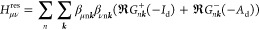
4where Green’s functions of the l-system describe electron transfer to (from) its bands |*n****k***⟩ and are given by
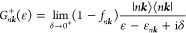
5
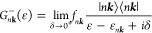
6In eqs 4–6, *I*_d_ and *A*_d_ are ionization potential and electron affinity of the d-system, β_μ*n****k***_ are resonance (hopping) integrals between μ-th d-orbital and *n****k***-th band of the l-system, and ε_*n****k***_ and *f*_*n****k***_ are energies and occupation numbers of the l-bands. Spin variables are omitted for clarity.

In summary, the periodic EHCF method^46^ represents the electronic structure of the solid system containing isolated TM atoms as s,p-band structure augmented with the d-multiplets. It accurately predicts the multiplicity and spin of the ground state and the energy of the low-lying excited d–d states.

### Calculation of the Mössbauer Spectrum

The wave functions and the energy of the ground and low-lying excited states obtained by the EHCF method can be used to calculate the parameters of the Mössbauer spectra,^54^ namely, the isomeric shift and quadrupole splitting. Here, we focus on calculations of the temperature dependence of the quadrupole splitting in the Mössbauer spectrum of periodic solids containing a radioactive isotope of iron, ^57^Fe.

In the electric field with the gradient represented by a tensor *V*_αβ_, the spin states of the nucleus split according to the following equation
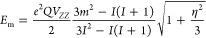
7where *V*_*ZZ*_ is the main component of *V*_αβ_, *Q* is the quadrupole moment of the nucleus, *I* is the nuclear spin, *m* is the projection of the nuclear spin in a given excited state, and η is the asymmetry parameter defined as
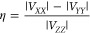
8Equation 7 shows that the states of the ^57^Fe nucleus do not split, while the excited states split into two levels corresponding to the values of the nuclear spin of 1/2 and 3/2. The energy difference gives the quadrupole splitting
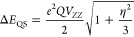
9

The tensor, *V*_αβ_, describing the gradient of the electric field acting on the nucleus of iron has three contributions.^55^ Two contributions, *V*_αβ_^4p/3d^, come from electrons of the p- and d-shells of the Fe atom, and they are described as
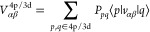
10while the third component of the tensor, *V*_αβ_^*L*^, originates from the charge density of the rest of the periodic crystal structure which is represented by a set of atomic point charges
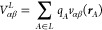
11where
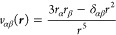
12In eqs 10–12, the iron nucleus is positioned at the origin, *P*_*pq*_ are the elements of the one-electron density matrix, *q*_*A*_ and ***r***_*A*_ are the effective charge and radius-vector of atom *A*, and *r*_α_ and *r*_β_ are the Cartesian components of the vector ***r***.

The core electrons, which are not included explicitly, yield the screening effect to the contributions from 4p- and 3d-orbitals [in reference to eq 13, the screening coefficient (1 – *R*) = 0.68] and the antiscreening effect to the contribution from ligands [coefficient (1 – γ_∞_) = 10.14].^56,57^ With these corrections taken into account, the electric field gradient can be expressed as
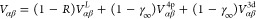
13Accounting for contributions of the excited electronic states populated according to the Maxwell–Boltzmann statistics gives the final equation for the electric field gradient
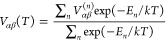
14This expression is used to describe the temperature dependence of the quadrupole splitting.

## Results and Discussion

### Carbodiimides and Hydrocyanamides of Transition Metals

**Figure 1:**
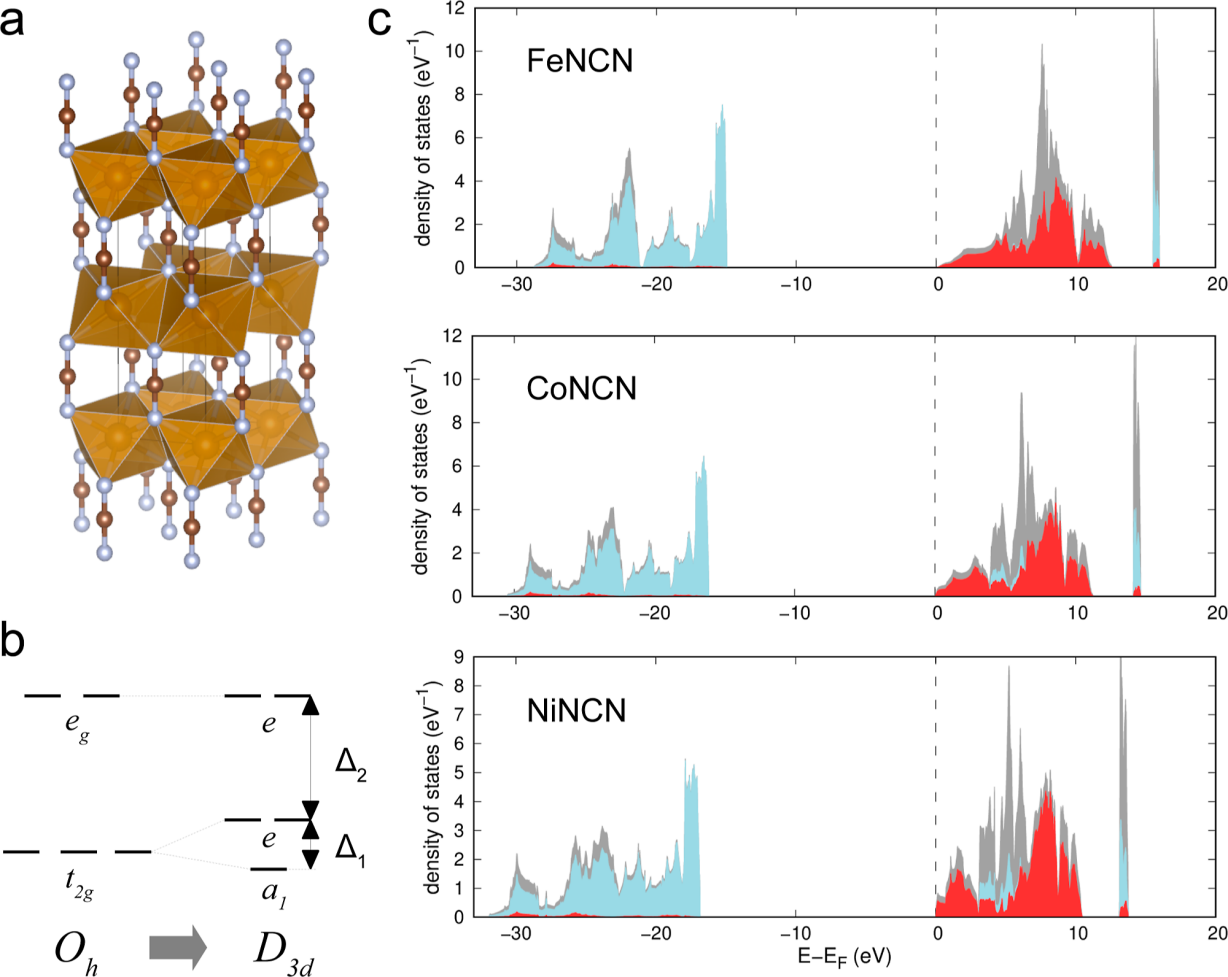
(a) Crystal structure of *M*NCN, red spheres correspond to *M*, blue spheres correspond to N, and brown to C; (b) diagram of the splitting of d-levels corresponding to the spatial *D*_3*d*_ symmetry of the coordination sphere of a TM ion; and (c) DOS of the l-subsystem: gray, blue, and red colors correspond to the total, N-, and *M*-projected density, respectively. Deep-lying s-states are not shown in the DOS plots for clarity.

**Figure 2:**
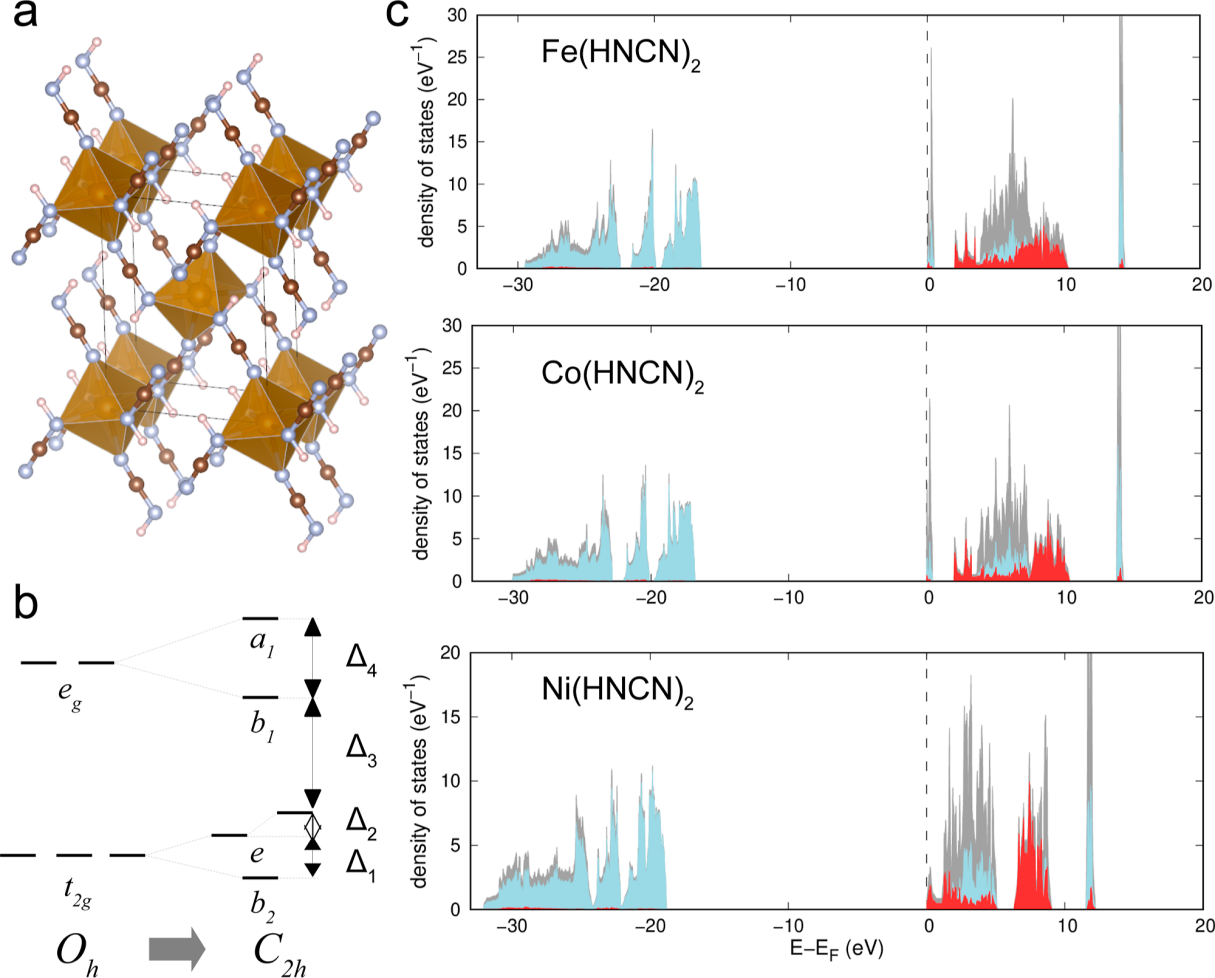
(a) Crystal structure of *M*(HNCN)_2_, red spheres correspond to *M*, blue spheres correspond to N, brown to C, and pink to H; (b) diagram of the splitting of d-levels corresponding to the spatial *C*_2*h*_ symmetry of the coordination sphere of a TM ion; and (c) DOS of the l-subsystem, as described in Figure 1.

Relatively simple and well-studied experimental structures of carbodiimides, *M*NCN, and hydrocyanamides, *M*(HNCN)_2_ (M = Fe, Co, Ni), have been selected for the initial test calculations. Figures 1 and 2 show the calculated densities of states (DOSs) for the l-subsystem of carbodiimides and hydrocyanamides, respectively. In all cases, the density of the occupied states near the Fermi level is almost completely determined by the nitrogen orbitals, while unoccupied states consist of a mixture of nitrogen orbitals and s,p- orbitals of TM, with the predominance of the latter. As nitrogen atoms form the nearest coordination sphere of the metal, the resonance contribution to the effective Hamiltonian of the d-system is mainly determined by the electron transfer states N(2p) → M(3d) and N(2s) → M(3d). This is analogous to the previous results for TM oxides,^46^ where electron transfer from oxygen orbitals to the vacant 3d orbitals was responsible for the resonance contribution. Another interesting similarity between *M*NCN and the corresponding oxides (*M*O) is manifested in very close values of the net atomic charge on metal ions (1.26, 1.23, and 1.22 for FeNCN, CoNCN, and NiNCN; 1.25, 1.24, and 1.23 for FeO, CoO, and NiO^46^). This means that the NCN^2–^ ion might be considered as an electronic analogue of the O^2–^ ion.

The observed local symmetry of the TM ion environment in carbodiimides is lower than that of the ideal octahedron due to trigonal distortion and distortions of the *M*–N bond length and N–*M*–N angles. This leads to an additional splitting of the *t*_2g_ level, as shown in Figure 1b; the corresponding splitting parameters are shown in Table 1. The splitting of the *t*_2g_ level is rather small, which reflects a slight deviation from the octahedral symmetry. The splitting parameter, Δ_1_, depends weakly on the resonance integrals, and it is mainly determined by the contribution from the crystal field interactions. Hence, Δ_1_ depends only on the effective charges of atoms obtained in the band structure calculations for the l-subsystem. At the same time, the splitting parameter Δ_2_ is essentially determined by the resonance interactions (resonance contribution is ca. 85% in all cases).

**Table 1:** Calculated Splitting Parameters of the d-Levels (Δ_1–4_, in eV) and the Spin of the Ground State (*S*) for a Series of *M*NCN, *M*(HNCN)_2_, and *M*-MOF-74^a^

	Δ_1_	Δ_2_	Δ_3_	Δ_4_	*S*	μ_theor_	μ_exp_
FeNCN	0.14	1.04			2	4.90	3.9^48^
CoNCN	0.13	0.89			3/2	3.87	3.7^49^
NiNCN	0.14	0.73			1	2.83	1.9^49^
Fe(HNCN)_2_	0.10	0.03	0.84	0.59	2	4.90	4.5^48^
Co(HNCN)_2_	0.09	0.03	0.75	0.39	3/2	3.87	
Ni(HNCN)_2_	0.10	0.03	0.84	0.33	1	2.83	
Fe-MOF-74	0.05	0.34	0.61	1.62	2	4.90	3.64^60^
Co-MOF-74	0.11	0.33	0.51	1.31	3/2	3.87	3.45^60^
Ni-MOF-74	0.10	0.26	0.70	1.08	1	2.83	3.36^60^

a The calculated magnetic moments (in μ_*B*_) are compared with the experimental literature values.

In hydrocyanamides, tetragonal distortions occur leading to a reduction from octahedral symmetry to the *D*_4*h*_ group. An additional distortion of the N–*M–*N angles leads to further lowering of the symmetry, so the final splitting diagram corresponds to an irreducible representation of the *C*_2*h*_ group as illustrated in Figure 2b. Therefore, the splitting of *d*-levels in *M*(HNCN)_2_ is described by four parameters presented in Table 1. In all cases, the ground state is a high spin state, which corresponds to the weak crystal field. The calculated spin-only values of the magnetic moment, which describe a magnetically diluted case, are also listed in Table 1. When comparing these values with experimental data,^48,49^ we must acknowledge that these systems are by no means magnetically diluted. Experiments on the temperature dependence of magnetic susceptibility^48,49^ demonstrate that these systems exhibit antiferromagnetic behavior with rather high Néel temperatures. This is caused by the exchange interactions between individual d-shells and leads to the lower experimental values of the magnetic moment compared to the computationally predicted values.^58,59^

The calculated energies of the d–d transitions are given in Tables 2–4. A comparison with previous EHCF calculations performed in cluster approximation^58^ shows that the solid-state periodic implementation of the method leads systematically to higher d–d excitation energies. This is in agreement with our previous conclusions regarding TM oxides.^46^ It is also interesting to compare the periodic EHCF results for the excited energies of carbodiimides with the periodic DFT calculations (GGA + U) performed in ref (59). In this work,^59^ it has been concluded that GGA + *U* yields the overestimated value for the band gap of NiNCN and CoNCN, which contradicts our knowledge of the simplest optical properties of these materials. Furthermore, for FeNCN, GGA + *U* predicts the ground state to be metallic, which is not consistent with experimental observations. From this point of view, the periodic EHCF method^46^ shows an improvement in our theoretical understanding of the optical properties of *M*NCN.

**Table 2:** EHCF Energy (in eV) of the d–d Excitations for Periodic FeNCN and Fe(HNCN)_2_ Structures as Compared to the Cluster EHCF Results^58^

FeNCN			Fe(HNCN)_2_		
symmetry	periodic	cluster	symmetry	periodic	cluster
^5^*A*_1_	0	0	^5^*A*_1_	0	0
^5^*E*	0.14	0.03	^5^*E*	0.10	0.05
	0.14	0.03		0.13	0.06
^5^*E*	1.17	0.83	^5^*E*	0.97	0.75
	1.19	0.95		1.56	1.13
^3^*E*	1.76	1.93	^3^*E*	1.34	1.79
	1.80	1.94		1.61	1.84
^3^*A*_1_	1.89	1.97	^3^*A*_1_	1.71	2.01
^3^*E*	2.05	2.25	^3^*E*	2.05	2.12
	2.18	2.27		2.11	2.23
^1^*A*_1_	2.09	2.46	^1^*A*_1_	1.73	2.33

**Table 3:** EHCF Energy (in eV) of the d–d Excitations for Periodic CoNCN and Co(HNCN)_2_ Structures as Compared to the Cluster EHCF Results^58^

CoNCN			Co(HNCN)_2_		
symmetry	periodic	cluster	symmetry	periodic	cluster
^4^*A*_1_	0	0	^4^*A*_1_	0	0
^4^*E*	0.04		^4^*E*	0.04	
	0.04			0.04	
^4^*A*_2_	0.85	0.70	^4^*E*	0.72	0.73
^4^*E*	0.77	0.72		0.80	0.74
	0.78	0.72	^4^*A*_2_	1.08	0.75
^4^*A*_2_	1.67	1.47	^4^*A*_2_	1.83	1.54
^2^*E*	1.65	1.81	^2^*E*	1.37	1.72
	1.65	1.81		1.67	1.79

**Table 4:** EHCF Energy (in eV) of the d–d Excitations for Periodic NiNCN and Ni(HNCN)_2_ Structures as Compared to the Cluster EHCF Results^58^

NiNCN			Ni(HNCN)_2_		
symmetry	periodic	cluster	symmetry	periodic	cluster
^3^*A*_2_	0	0	^3^*A*_2_	0	0
^3^*E*	0.76	0.72	^3^*A*	0.69	0.73
	0.76	0.74	^3^*E*	0.84	0.84
^3^*A*	0.69	0.87		0.99	0.85
^3^*E*	1.21	1.26	^3^*A*	1.32	1.33
	1.22	1.33	^3^*E*	1.45	1.40
^3^*A*	1.37	1.48		1.48	1.44
^1^*E*	1.90	2.05	^1^*E*	1.91	2.10
	1.91	2.11		1.97	2.11

**Figure 3:**
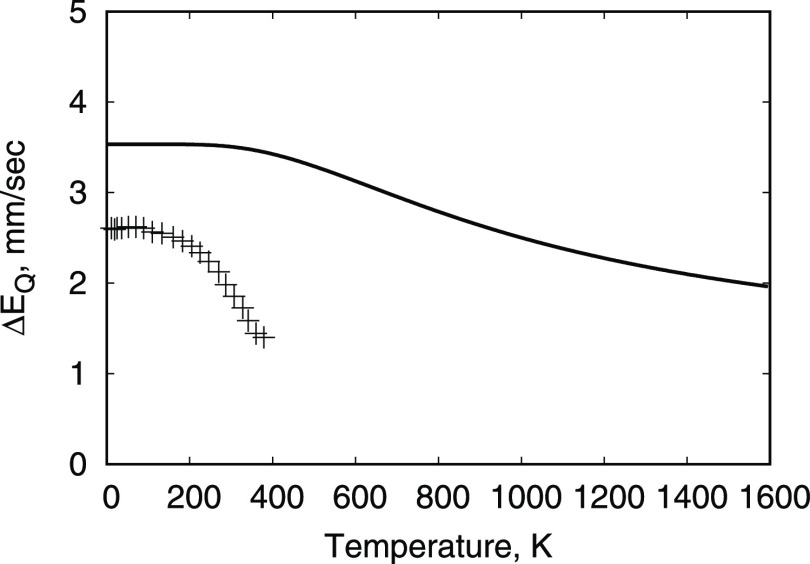
Temperature dependence of the quadrupole splitting Δ*E*_Q_ in the Mössbauer spectra on ^57^Fe nuclei for FeNCN. Crosses indicate the experimental values,^61^ and the line corresponds to the EHCF values.

As no detailed experimental data are available on the optical spectra of *M*NCN and *M*(HNCN)_2_, it makes it impossible to readily validate the predicted energies of *d*–*d* transitions. However, the temperature dependence of the quadrupole splitting in the Mössbauer spectrum of ^57^Fe nucleus is known from experiment for FeNCN,^61^ which allows us to test the accuracy of our predictions for the energy of the first excited state, ^5^*E*, relative to the ground state of *D*_3*d*_ symmetry. Figure 3 shows a comparison of the experimental Δ*E*_Q_ temperature dependence with the calculated result, and it indicates that, in agreement with experiment, the calculated values of Δ*E*_Q_ decrease with increasing temperature, although the temperature scale and the splitting scale are overestimated. The temperature scale is determined by the energy of the first excited state ^5^*E*. The correct scale of the temperature dependence corresponds to the energy of the first excited state equal to 0.06 eV, while the EHCF calculation gives 0.14 eV. Note that the relative energy of the ^5^*E* state is determined only by the parameter Δ_1_ and it does not depend on the two-electron interactions. At the same time, as mentioned above, Δ_1_ depends only on the charges on the atoms. Therefore, we conclude that the source of the discrepancy seems to be associated with the choice of the parametrization scheme used to calculate the band structure of the l-subsystem. It is well-known that the CNDO/2 scheme typically overestimates the charges compared to other approaches, for example, the SINDO1 scheme^62^ so that this shortcoming can be easily eliminated by selecting a more appropriate parametrization.

For Fe(HNCN)_2_, the quadrupole splitting was measured for one value of the temperature only, namely, *T* = 300 K.^61^ These experimental results suggest that for this compound, the d-orbital does not undergo a rapid relaxation resulting in two observed values of 1.85 and 2.67 mm/s. The EHCF calculations assume that if this relaxation takes place, it gives the value of 3.21 mm/s. For the three lowest energy states of the d-shell, the values of the quadrupole splitting are 3.31, 3.39, and 2.39 mm/s. These results show a proportional overestimation of about 25%, which is consistent with the data obtained for FeNCN.

### *M*-MOF-74 Metal–Organic Frameworks

**Figure 4:**
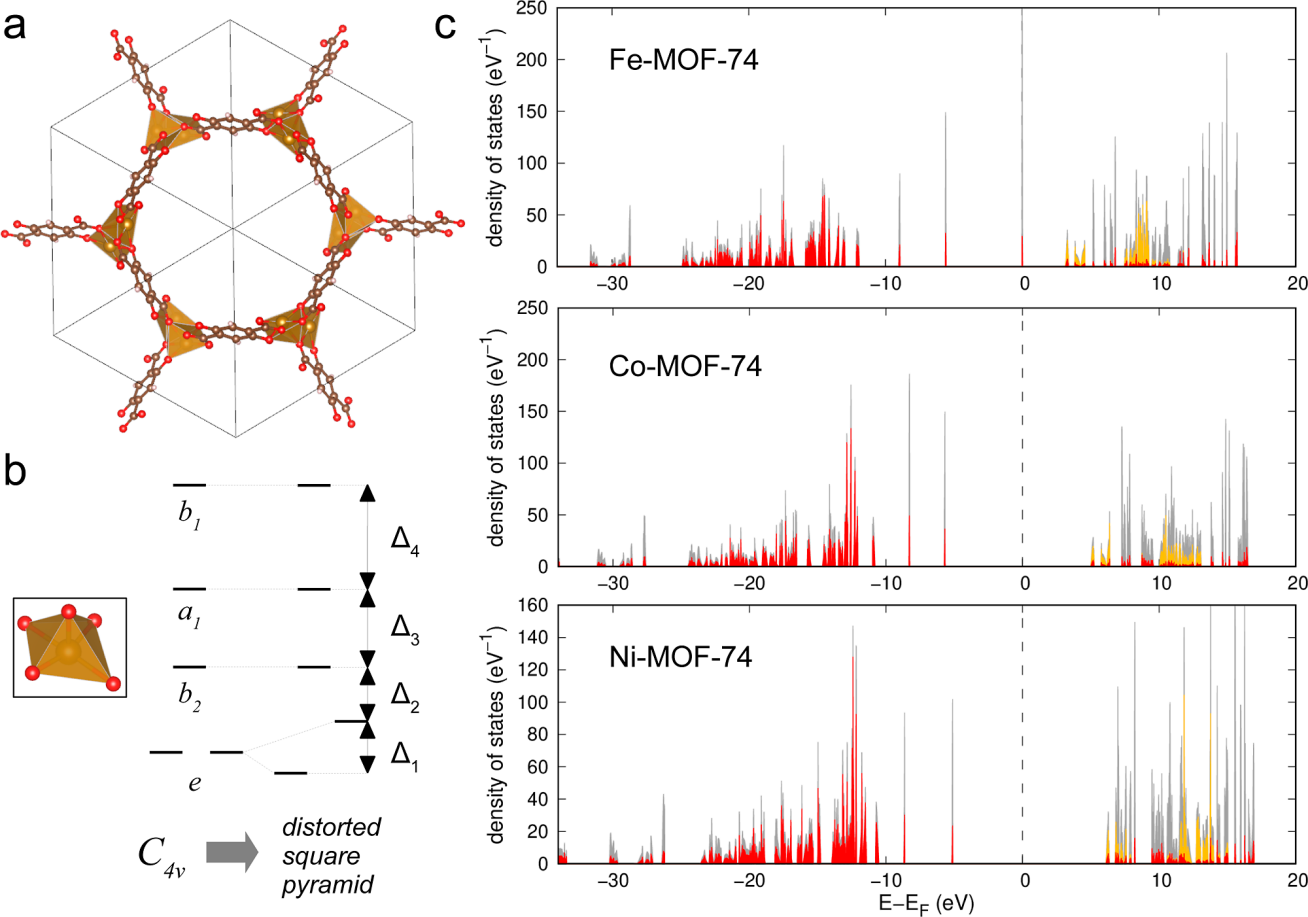
(a) Crystal structure of *M*-MOF-74, gold spheres correspond to *M*, red spheres correspond to O, brown to C, and pink to H; (b) metal atom coordination polyhedron in *M*-MOF-74 and splitting of the *d*-levels corresponding to the spatial *C*_4*v*_ symmetry of the TM ion coordination sphere; and (c) DOS of the *l*-subsystem: gray, red, and gold colors correspond to the total, O-, and *M*-projected density, respectively. Deep-lying s-states are not shown in the DOS plots for clarity.

The structure of *M*-MOF-74 and the calculated DOS are shown in Figure 4. In all cases, DOS consists of sharp narrow peaks, which reflect a small degree of delocalization between organic linkers of the frameworks. The coordination sphere of the TM ion is a distorted tetragonal pyramid approximately corresponding to the *C*_4*v*_ point symmetry group. Therefore, the splitting of one-electron d-states corresponds to the diagram depicted in Figure 4b. The values of the splitting parameters are listed in Table 1.

The calculated magnetic moments of the ground state and d–d transitions are presented in Tables 1 and 5. In all cases, the calculated ground state of the d-shell is high-spin, which agrees with experiment^60,63^ and previous hybrid MP2/DFT-D calculations.^42^ Due to the close proximity of TM atoms in MOF-74, the magnetic interaction between them (antiferromagnetic for Fe-MOF-74 and Co-MOF-74 and ferromagnetic for Ni-MOF-74) leads to a significant deviation of the experimental value for the magnetic moment from the pure spin values. For Fe and Co, we overestimate the magnetic moment, similar to the case of carbodiimides, whereas the ferromagnetic behavior of Ni-MOF-74 leads to a higher experimental value compared to the computational prediction.

**Table 5:** EHCF Energy (in eV) of the d–d Excitations for the *M*-MOF-74 Frameworks

Fe-MOF-74		Co-MOF-74		Ni-MOF-74	
Spin	energy	spin	energy	spin	energy
2	0.05	3/2	0.18	1	1.09
2	0.39	3/2	0.27	1	1.19
1	0.66	1/2	0.76	0	1.40
1	0.86	3/2	0.99	1	1.77
1	0.95	3/2	1.35	1	1.78
2	1.00	3/2	1.51	0	1.95
0	1.01	1/2	1.75	1	2.53

## Computational Details

The calculations performed in this work employ NDO-type parametrization^43^ with the resonance parameters originally fitted for molecular complexes.^64^ MOF structures were taken from the Cambridge Structural Database (CSD).^65^ The band structure of the l-subsystems was calculated using the periodic Hartree–Fock method on the basis of local atomic orbitals with the Ewald summation for the long-range Coulomb integrals, (11 × 11 × 11) Monkhorst–Pack *k*-mesh for *M*NCN and *M*(HNCN)_2_, and (5 × 5 × 5) for *M*-MOF-74. These calculations take ca. 8–10 h on a 12-core processor, and the time-limiting step is the evaluation of the band structure of the extended l-subsystem. Spin-only values of the magnetic moments of TM atoms were calculated using the following equation
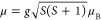
15where *S* is the spin of the ground state of the d-shell and *g* ≈ 2 is the electronic Landé factor.

## Conclusions

The computational algorithms based on semiempirical implementation of the periodic EHCF method have been developed and described in ref (46). In this work, we have extended the applicability of the method to include its performance improvements for large unit cells and to add the calculations of the parameters for the Mössbauer spectrum. A particular emphasis has been placed on the efficiency of the parallel implementation of the code, which was significantly improved.

The hybrid QM/QM EHCF method^43,45^ extended to periodic systems^46^ has been applied to study the electronic structure of TM carbodiimides and hydrocyanamides and of *M*-MOF-74 frameworks of Fe, Co, and Ni. The multiplicity of the ground state and the experimentally observed magnetic moments have been reproduced accurately for all of the considered chemical systems. A comparison with the experimental data on the Mössbauer spectrum of FeNCN and Fe(HNCN)_2_ has demonstrated the ability of the EHCF method to reproduce the spatial symmetry and energy of the low-lying excited *d*-states with very reasonable accuracy. The EHCF calculations are computationally effective and free from convergence problems typical for DFT methods; this makes EHCF a reliable method for numerical modeling of the electronic structure and magnetic and optical properties of MOFs.
